# Conducting ethical drug research in prisons: lessons learnt from the implementation of the European Questionnaire on Drug use among People living in Prison (EQDP)

**DOI:** 10.1108/IJOPH-05-2025-0039

**Published:** 2026-02-02

**Authors:** Linda Montanari, Els Plettinckx, Arkadiusz Komorowski, Nadine Berndt, Liesbeth Vandam, Daan van der Gouwe, Grainne Cousins, Josefina Mavrou, Ioulia Bafi, Mika Rautanen, Tanja Schwarz, Luis Royuela, Marie Claire Van Hout

**Affiliations:** European Monitoring Centre for Drugs and Drug Addiction, Lisbon, Portugal; Sciensano, Brussels, Belgium; Department of Psychiatry and Psychotherapy, Medical University of Vienna, Vienna, Austria, and the Grüner Kreis Association, Vienna, Austria; Department of Epidemiology and Statistics, Directorate of Health Luxembourg, Luxembourg, Luxembourg; European Monitoring Centre for Drugs and Drug Addiction, Lisbon, Portugal; Trimbos Institute, Utrecht, The Netherlands; Royal College of Surgeons in Ireland, Dublin, Ireland; Cyprus National Addictions Authority, Nicosia, Cyprus; University Mental Health Research Institute, Athens, Greece; THL, Helsinki, Finland; Addiction Competence Centre, Vienna, Austria; European Monitoring Centre for Drugs and Drug Addiction, Lisbon, Portugal; Office of the Vice President of Research, Innovation and Impact, South East Technological University, Waterford, Ireland

**Keywords:** Prison, Guidelines, Europe, Drug addiction, Ethic, Research

## Abstract

**Purpose:**

This paper aims to provide a practical tool to guide researchers in conducting drug research in prison, outlining the main research phases − preparation, methodological choices, implementation and ethical approval − as well as challenges and recommendations for each phase.

**Design/methodology/approach:**

This paper is based on the experiences of implementing the European Questionnaire on Drug use among People Living in Prison, a model questionnaire developed by the European Union Drugs Agency, in some European countries.

**Findings:**

During the research preparation, objectives, map of the prison system, understanding of legislation and context and key actors should be identified. A formal request should be sent to the prison authorities. Methodological decisions include choice of data collection tools, sampling strategies, implementation and dissemination. Flexibility is essential in implementation, while ensuring independence from prison staff. Adequate equipment, space and feedback to participants are recommended. Ethical approval is critical.

**Research limitations/implications:**

This paper is based on the experience of some European countries, and other barriers and facilitators can be found in other contexts.

**Practical implications:**

This paper describes the barriers to conduct research in prison and the possible solutions to overcome them, providing a practical tool to conduct ethical drug research in prison.

**Social implications:**

This paper can have profound social implications helping researchers across Europe to identify health and social needs of this population.

**Originality/value:**

This paper provides a unique insight for strategies to overcome challenges related to the implementation of ethical drug research in prisons, laying a foundation to support harmonised drug research in prison across countries.

## Introduction

In 2022, there were 11.5 million people living in prison (PLIP) on any given day worldwide and around one million in Europe (Aebi and Cocco, 2024; Walmsley, 2024). Around one in five persons in prison are detained for drug offences (Aebi and Cocco, 2024). People living in prison are disproportionately affected by poor health and burden of diseases, including mental and drug use disorders as well as infectious diseases (e.g. HIV and hepatitis C) [Busschots *et al.*, 2022; Dolan *et al.*, 2016; Favril *et al.*, 2024; Fazel and Seewald, 2012; Fazel *et al.*, 2016; World Health Organization (WHO) – Regional Office for Europe, 2023]. Compared to the general population, high levels of lifetime prevalence of illicit drug use are documented in the European prison population [Van de Baan *et al.*, 2021; European Monitoring Centre for Drugs and Drug Addiction (EMCDDA), 2022]. Whilst many individuals are reported to cease using drugs inside prisons, some continue or initiate to use drugs, with high health risk practices (i.e. use of new psychoactive substances or sharing drug injecting equipment) [European Monitoring Centre for Drugs and Drug Addiction (EMCDDA), 2018; European Monitoring Centre for Drugs and Drug Addiction (EMCDDA), 2022; Van de Baan *et al.*, 2021]. Services for people with drug use experience exist inside European prisons, although their coverage remains limited compared to the community (such as opioid agonist treatment and needle-syringe programmes) [European Monitoring Centre for Drugs and Drug Addiction (EMCDDA), 2022].

Knowing and addressing the health needs of the prison population has public health benefits for the wider community, as PLIP represent a dynamic population in regular contact with the community [European Monitoring Centre for Drugs and Drug Addiction (EMCDDA), 2022]. The importance of health care and research in prison is recognised in the European Union’s Drug Strategy and Action Plan 2021–2025 (European Union, 2021; Stöver *et al.*, 2020).

Data on drug use among PLIP is scarce, as the prison environment poses substantial challenges for conducting drug research. For that reason, the European Union Drugs Agency (EUDA), which is the central authority on illicit drugs in the European Union, supporting the European Member States to anticipate, alert, respond and learn on how to address drug problems, has developed a research instrument to monitor the drug situation in prisons: the European Questionnaire on Drug use among people living in Prison (European Drugs Agency, 2025a, 2025b) (European Union, 2021; European Union, 2023). The EQDP is a model questionnaire that includes questions in the areas of: socio-demographic and judicial status, substance use, injecting and other health risk behaviours, health status, use of health and addiction services, social reintegration (Montanari *et al.*, 2024, 2025; Royuela *et al.*, 2025), and can be used for quantitative research (Montanari *et al.*, 2024, 2025; Royuela *et al.*, 2025). As conducting research and monitoring drugs and prisons is sensitive and challenging, it is important to provide practical guidance for conducting ethically sound and robust studies in prison [Ahalt *et al.*, 2018; Ako *et al.*, 2020; Council for International Organizations of Medical Sciences (CIOMS), 2016; Creswell, 2014; Dalen and Øen Jones, 2010; Eldridge *et al.*, 2011; Gill *et al.*, 2023; McDermott, 2013; Mhlanga-Gunda *et al.*, 2019; Moser *et al.*, 2004; Pont, 2008; Sánchez, 2012; Shaw, Wangmo and Elger, 2014; World Medical Association (WMA), 2018]. Notably, specific attention needs to be paid to overcome the fear of PLIP to provide answers on drug use. Considering that data collection should focus on public health issues and not on control by the penal authorities, a survey has to be managed by independent institutions/persons to overcome reluctance about participating in the study. Adaptation to differences in legal and cultural contexts has also to be considered.

Therefore, this paper aims to provide practical tools to support researchers in conducting drug research in prisons, by describing the phases of research and the challenges pertaining to each phase, including research preparation, methodological choices and ethical approval.

The implementation of the EQDP questionnaire [1] (or its adapted versions) in the countries of the European Union has proven to be an extremely useful tool to increase data collection in that area, as demonstrated by the substantial increase in the number of surveys carried out in detention establishments, especially between 2020 and 2023. Comparing the periods 2020–2023, 2016–2019 and 2012–2015 the number of surveys increased threefold (European Drugs Agency, 2025a, 2025b). Adhering to the EQDP methodological guidelines [2] some principles should be at the basis of the implementation of prison and drugs surveys: data collection focus on public health and not on control, it is highly recommended the survey be managed by institutions/persons independent from prison system to overcome reluctance and fear about participating in the study. The questionnaire can be adapted to fit the prison site/country context and combined with qualitative interviews to gain additional insights from participants. Whilst certain questions can be dropped or added, it is advisable to keep the wording, length and order of questions intact to facilitate possible future comparisons between different contexts or countries. The instrument is complemented by methodological guidelines (Montanari *et al.*, 2024, 2025), covering the key principles, guidelines, definitions for each question and specifications for conducting drug research in prison; it also provides recommendations regarding periodicity, geographical area and sampling. The EQDP (or its adapted versions) was implemented in several European countries between 2014 and 2023, using different languages according to the populations living in prison (Belgium, Cyprus, Finland, Greece, Latvia, Lithuania, Luxembourg, Poland, Portugal and Spain) (Mazzilli *et al.*, 2022; Montanari *et al.*, 2024, 2025; Plettinckx *et al.*, 2024). The samples of the surveys that have been carried out so far vary between 140 and 5,600 PLIPs and the response rate between 24% and 87% (Table 1).

**Table 1:** EQDP, or adapted versions, surveys carried out in EU countries, 2018–2023

Country	Sample	Year	Response rate (%)
Belgium	288	2021	75.5
Cyprus	141	2022	n.a*.
Finland	529	2022	82.5
Greece	2,131	2022	24.0
Latvia	1,178	2022	58.5
Lithuania	448	2021	68.9
Luxembourg	193	2022	32.0
Portugal	2,051	2023	66.4
Poland	1,251	2018	n.a.
Spain	5,512	2022	86.4

Note(s):

*The response rate of the survey conducted in Cyprus cannot be determined, as inmate participation was voluntary and the survey was conducted within the framework of the prison school programme. As such, the sample was not randomly selected, and attendance in educational settings may not reflect the broader prison population

## Method

The method of this study is a structured consultation methodology, grounded in participatory action research and integrated knowledge translation principles. The methodology is based in three complementary components, as follows.

Firstly, expert panel sessions were held with invited prison experts, who met during two days in Cyprus in 2023 to discuss predefined key topics related to conducting drug research in prison settings, followed by moderated discussions and Q&A. A rapporteur synthesised key messages from each meeting, identifying areas, actions and task for conducting drug research in prison.

Secondly, the European prison and drug research working group coordinated by the EUDA[3] convened regularly to review progress, discuss key topics related to research and integrate expert insights into ongoing research and implementation activities.

Thirdly, the experience in carrying out prison survey with the model questionnaire – EQDP – contributed to identify priority areas, actions and key tasks for carrying out drug research in prison. The EQDP methodology focuses on public health and its aim is to collect information that can be used to improve health, social services and facilities for people living in prison and ultimately to improve the physical, psychological and social conditions of people living in prison; this should improve the health of the whole community. Also, the EQDP has the added value to be the same (or similar) in different countries, therefore allowing data comparability on one hand and inspiring the adoption of best practices across different countries.

This multi-source, iterative process ensured that the research was evidence-informed, context specific and refined through targeted expert input and iterative continuous stakeholder engagement.

Four key phases and main challenges in conducting drug research in prison were identified. For each key phase, a country specific case is described. Three of these countries participated in the PRS20 project (Plettinckx *et al.*, 2024), a European project aiming to assess the needs of PLIP and ex-PLIP based on the implementation of the EQDP as well as the design of tailor-made interventions to reduce drug use in prisons and address new threats. Finland has carried out a prison study in 2022.

Notably, four countries demonstrated a specific focus and best practice in dealing with different phases of research implementation; and in particular: accessing prison (Cyprus), participation rate (Finland), mode of administration (Belgium), data protection (Luxembourg) (see Box 1–4). Their activities and solutions identified for each phase of the research can be useful good examples for other countries who want to implement prison and drug research.

## Results

The first phase – *preparation and planning* – includes the definition of the research questions and their specific objectives. It is important to ensure that those are pertinent to the local prison context, the access to prison, the identification of adequate human resources and the logistics of conducting research in a closed setting.

A preliminary mapping of the prison system in the country should be conducted to understand the overall research context. Particular attention should be paid to the legislative framework, especially concerning national drug law, number and types of prison establishments and regimes, organisational and logistic structures and security measures. In relation to the legislative framework, special attention needs to be paid to prison laws and internal regulations (disciplinary and criminal sanctions) regarding drug possession and use of drugs within prison, such as temporary loss of privileges such as temporary leaves, external work or conditional release and how these measures might impact the study, especially concerning information on drug use within prison.

It is also crucial to identify the key actors inside prison. It is necessary to write a formal request letter to the prison authorities, including information on research protocol, EQDP survey and other research material relating to survey as well as the logistics of research implementation.

Researchers should be aware of the timing involved in obtaining permissions to access prison, which might be substantially long. Preparation should start well in advance, ideally one or two years before the date foreseen for the actual data collection. A research protocol should be developed, as a basis for obtaining ethical approval and security clearances. The protocol should be as specific as possible and emphasise the public health scope of the research, which must benefit all people living and working in prison, including PLIP and prison administration.

Sufficient and adequate financial and human resources should be made available to support and conduct the study. It is important to select researchers with a good understanding of sensitive issues and delicate dynamics of the prison. Researchers from local universities, research institutes or other institutions can take part in the study; in some cases health and social care prison staff can contribute to the study. Each team member should be attributed with clear roles and must be adequately trained on potential difficult situations related to safety; at the same time, they must be sensitised to avoid instances of discrimination and stigma. Researchers should be sufficiently trained on scientific standards, roles and ethical considerations and security protocols in conducting research in prisons. It is preferable not to contract inexperienced researchers, who may be psychologically vulnerable to difficult contexts and hard life experiences, which will inevitably emerge during the research. An independent, professional attitude is paramount, whilst a moralistic approach is not only unacceptable in this context, but also counterproductive for the research’s objectives. Any potential conflicts of interest, such as research results censorship by prisons’ administration or prison authorities preventing PLIP from participating in the survey, with prison authorities must be disclosed, and dealt with accordingly (Simpson *et al.*, 2025).

It is also advisable to include people with lived experience in the research team, which may reduce barriers in PLIP’s involvement in the research, increasing trust in the researchers, and enhancing research credibility regarding findings on both drug use outside/inside prison and prison experience (Moser *et al.*, 2004).

The preparation of the logistics for the entry into prisons is a key part of the preparatory phase and should take into consideration the specific prison conditions, including the restricted access to internet or limitations in materials, space and time inside prison. It is finally necessary to establish in advance which languages are spoken inside prison with EQDP tools prepared accordingly (e.g. information flyers, informed consent and questionnaire).

**BOX 1 – Cyprus: accessing prison and preparing the research**

Cyprus conducted the first research on drugs and prison in the country in the framework of the PRS20 project (Plettinckx *et al.*, 2024), a EU funded project aimed at improving drug interventions inside prison, coordinated by Cyprus. Access to the Cyprus Prison Department was negotiated through the Ministry of Justice, the Public Order and the local prison directors, and the EQDP project was approved by the Cyprus National Bioethics Committee (CNBC). To gain access to the prison system, it was essential to include the Ministry of Justice, specifically the Prison Department, as a partner in the project. Once the Prison Department agreed to participate, several meetings were organised to discuss the project. The entire process of organising and achieving access to the prison and preparation of the EQDP study took 18 months. The EQDP was adapted to the Cypriot context and was translated into Greek, English and French. The response rate of the survey conducted in Cyprus could not be determined, as PLIP participation was voluntary and the survey was conducted within the framework of the prison school programme. As such, the sample was not randomly selected, and attendance in educational settings may not reflect the broader prison population.

The second phase of research on prison and drugs concerns the decisions on *methodological choices*. The EQDP methodological guidelines recommend the use of a mixed quantitative and qualitative method, where the two sources of information are combined to gain more insight into the topic. Specific attention should be given to the sampling framework and the mode of administration. Random sampling is the preferred method; the sample should be obtained from an anonymous list of people living in prison (PLIP) at the time of the research. Depending on its objectives, the EQDP study could target the entire prison population, a representative sample or subgroups. Additional considerations regarding sampling frame should recognise the presence of some groups of PLIP, who may not be captured by random sampling procedures, such as women, LGBTQIA+ people, persons with disabilities, foreign nationals, forensic psychiatric patients and others (Koch *et al.*, 2023). It is important that the prison administration does not influence the choice of the sample, although it can indicate who is unable to participate in the study or specific reasons which should be made explicit to the researchers (e.g. illness and hospitalisation, solitary confinement, mental or cognitive problems and level of literacy). This is an essential step to manage the research. Regarding the method of administration, the EQDP guidelines give alternative options, including self-administration, face-to-face interviews or computer-assisted interviews.

To facilitate participation and increase response rate, the research should be introduced to the potential participants by clearly describing the research objectives of the EQDP, the data collection and analysis process and the dissemination of the results. During the introduction it should be stressed that the information will remain strictly confidential and anonymous and only the researcher will have access to it. Information flyers or posters, available in different languages, may be distributed a few days in advance to inform PLIP about the study. While incentives for participating in prison research may be used, those incentives should not include objects or goods that may put participants at any security and health risk or ethical dilemmas.

**BOX 2 – Finland: increase participation rate in the study**

Prison research conducted in Finland is a useful example for sound methodological choices and especially high response rate. Finland conducted a large population health examination study concerning PLIP in 2022 complemented by interviews, health examinations, X-ray and laboratory tests. An advertisement campaign with slogans and graphical posters was launched and PLIP were made aware of the study beforehand. The research team created posters with colourful pictures and slogans such as *Empowering the prisoner* and disseminated them in the prisons’ corridor walls to sensitise the prison community around the upcoming study. Different kinds of encouraging comments and recommendations were strategically placed in between the subdivisions of question patterns. As a result, the participation rate was as high as 82.5% and they expressed willingness to participate voluntarily although participants were chosen at random.

The third phase is the actual *research implementation*. A flexible research approach is recommended. The study team should become familiar with the in-prison time schedule in advance of data collection. Prisons have a rigid time schedule from wake up to sleep, including specific hours for meals, locked cells, open air hours, work, sport activities, educational activities and others. Delays relating to security may occur; simple tasks potentially take longer inside prisons than outside. In principle, custodial staff should not directly be involved in research implementation, so as to respect the privacy and confidentiality of the participants. It is very valuable that researchers have an active role during the implementation phase of the research, implying that researchers have direct contact with PLIP and prison staff. Specifically, in relation to PLIP, it is extremely important that the researchers themselves can explain the study objectives and its implications in line with the informed consent. This will support the building of a trusting relationship and overcome distrust among both PLIP and prison staff.

Involvement of prison staff is however required to support logistics and facilitate participation when research teams enter the prison. Custodial staff may collect participants from their cells and staff or may be busy in other activities during the research. Weekends may be a preferable time for administering the study, as participants are usually not committed to any activity, however challenges may exist with regard to reduced capacity of custodial staff to support logistics. The research should consider giving everyone the possibility to participate in the survey by considering their prison time schedule as much as possible. However, the feasibility of the survey should be prioritised, as often prisons are only accessible in certain hours.

Research teams should arrive at the prison adequately equipped for conducting the survey (e.g. tablets, pen/paper, translated materials), and be prepared for last-minute changes in time or location. It is essential to consider whether available survey software allows for offline completion as the internet is generally not available in prison. Feedback should be exchanged on a regular basis among the research team during data collection. *Ad hoc* barriers to a smooth research implementation may also arise at any time; for example, staff strikes or internal emergencies.

An adequate space should be made available, according to the modalities of the study implementation (i.e. individual face to face, small groups and computerised). Preferably, the questionnaire should be filled out in a designated space, especially if there are no single cells in the prison. If the questionnaire is self-administered in a group setting, the space should be large enough to ensure that there is no contact, exchange of opinions or discussions between survey participants, whilst for individual face-to-face administration smaller rooms can be used.

**BOX 3 – Belgium: using the most appropriate mode of administration**

In Belgium, the EQDP survey was administered using an offline survey app on electronic tablets, which enhanced confidentiality measures and autonomy. The questions were presented gradually on the screen and automatic consistency checks executed, allowing inconsistencies to be corrected. The questionnaire was provided to groups of 5–15 people in a separate room, where prison staff were not present. PLIP found this mode of administration also entertaining and might not be very expensive. Structured face-to-face interviews were also conducted in cases where literacy and communication challenges were observed. Data entry and cleaning was supported by the survey software on the tablets, with each record corresponding to an individual respondent. Quality and consistency checks to inspect for multiple and partial responses were performed, yielding higher quality data.

The last phase (fourth) to consider is the *ethical approval*. It is of utmost importance that the EQDP study is carried out in a way that promotes its potential benefits for PLIP, regarding their health and social needs. In the introduction the potential benefits should be clearly explained to the PLIP. Collected data should be only used for the research purposes during the conduct of the survey. Conducting drug research in prison in an ethical way is ensured by alignment of the (adapted) EQDP protocols to international guidelines, including the Helsinki Declaration, the International Ethical Guidelines for Biomedical Research involving Human Subjects and the CIOMS guidelines for epidemiological studies (CIOMS, 2016; WMA, 2018). The approval is obtained from the relevant ethical committee for conducting research in prison in each country. If such committee does not exist at the prison, local or national level, the ethical committee of the university or institution coordinating or supporting the research should be requested to provide the ethical approval for conducting the research. According to research ethics, the participation in the research must be voluntary and the researcher should obtain a true informed consent in the participation, having in mind the objective to balance direct benefits with potential risks; furthermore PLIP must have the opportunity to participate in research, ideally co-designed by them [CIOMS, 2016; Creswell, 2014; Dalen and Øen Jones, 2010; Eldridge *et al.*, 2011; Moser *et al.*, 2004; Pont, 2008; World Medical Association (WMA), 2018].

Practical recommendations (for all four research phases) are provided in the checklist of the supplementary material; for the ethical standards they are summarised in the Table 2 of the text, as follows.

**Table 2:** Adherence to ethical standards

Area	Actions/tasks to check
Set up ethical standards	Rely on: International guidelines including Declaration of Helsinki; the International Ethical Guidelines for Biomedical Research involving Human Subjects and the CIOMS guidelines for epidemiological studies General Data Protection Regulation
Promotes potential benefits for the people living in prison	Ensure that the people are protected from any direct or indirect disadvantages as consequences of participation Inform people living in prison of their rights Ensure that researchers ensure to inform people on the results of the survey Include recommendations that aim to benefit people’s lives inside prison in the final research results
Obtain ethical approval	Obtain approval from the ethical committee for conducting research in prison in your country If such a committee does not exist, obtain ethical approval from the university or the institution under which the research is conducted
Information should be anonymous	Ensure that the questionnaire is filled in a fully anonymous way
Ensure agreement of participants	An informed consent should be obtained before starting the survey A template is available
Survey should be totally voluntary	Explain participants that they can refuse from participation at any stage of the research without any consequence

Information prior to seeking informed consent should be imparted in written and verbal format to ensure the participant’s agreement and their full awareness of research objectives and content, study procedures including questionnaire distribution and collection, possible risks or inconveniences, benefits, guarantees and protection of privacy and data management protocols.

PLIP must be advised that they can refuse or withdraw from participation at any stage of the EQDP study without any consequence and must be protected from any direct or indirect harms as consequences of their participation in the research. Custodial staff should not be able to identify respondents or view/listen to respondents’ answers (depending on the research method), although the presence of custodial staff might be necessary for security reasons.

**BOX 4 – Luxembourg: respecting data privacy and data protection**

In Luxembourg the study was prepared for implementation at one eligible closed prison of the Grand-Duchy of Luxembourg; it was approved by the National Research Ethics Committee, which guaranteed respect for the rights of participants on information, free choice to participate/refuse/withdraw from the study. The study procedures ensured the anonymity of respondents, as no data allowing for the identification of an individual were shared. The questionnaire was self-administered with the option to refuse or request assistance. An informed consent was provided to participants who had to sign it before completing the EQDP. The form included information on study objectives and expected outcomes, respondents’ contribution, procedures to ensure confidentiality, distribution and collection of questionnaires, benefits and risks in study participation, protection of privacy and invitation to participate in an optional follow-up interview. The informed consent form included information on data processing and was done under the conditions provided in Regulation (EU) 2016 / 679 of the European Parliament and of the Council of 27 April 2016 on the protection of natural persons with regard to the processing of personal data, repealing Directive 95 / 46/EC. The form is freely available in the EQDP package [4].

## Discussion

Despite the strong evidence of a close interconnection between prison and drugs, research in this area remains underdeveloped and further efforts should be made to improve evidence on health and social needs of the prison population in relation to drugs. Conducting drug research in prison is essential to support the implementation of adequate health and social services for people in prison with drug problems.

The challenges of conducting drug research in prison should be considered from the beginning to the conclusion of the research.

This paper provides a unique insight into recommended strategies to overcome challenges related to the implementation of ethical and robust drug research in prisons. It lays a foundation to support harmonised research in prison across countries and on the practical experience of several European countries that have conducted drug research in prison.

In relation to the *preparation and planning*, it is essential to define a transparent time schedule for the research. A contingency management plan may be defined in advance, detailed in the research protocol and agreed with the prison authorities prior to implementation. The independence of the researcher(s) from the prison authorities is a key aspect of conducting ethical drug research in prison; the prison administration must not influence the choice of people to be sampled. The research should follow scientific and ethical standards.

Next, it is important to define the *research method* deemed most appropriate to the specific context. The checklist in the supplementary material (and the EQDP guidelines) represents a detailed, practical and step by step tool for choosing the valid and appropriate method for the specific prison context where the research will be implemented (Montanari *et al.*, 2024, 2025). Generally, a mixed-method approach (quantitative and qualitative) is recommended, which allows triangulation across information sources (Creswell, 2014).

In the *implementation* phase, the internal prison organisation, the available space for conducting research in prison and the equipment necessary to conduct research should be prepared carefully. It is also important to address *ad hoc* barriers which might emerge during the research implementation and prepare to provide feedback to PLIP after the research field; this would allow a further validation of the data collected in prison.

The adherence to *ethical principles* was identified as the fourth phase since respect of high ethical standards is fundamental in the prison context involving highly vulnerable populations. Several indications, practical advice and tools are provided in the text and in the supplementary checklist, including, among others, approval from an ethical committee, use of the informed consent and adherence to international guidelines.

An important issue emerging from the survey is the extent of reporting on drug use within prison. Based on the experience of the countries carrying out the surveys in prison settings, both, the risk of people who refuse to participate and socially desirable responses always exist, as well, people who would lie tend to deny participating in the research, although there are differences between countries according to their cultural, legislative and prison context. Nevertheless, results from the surveys show that PLIP who agree to participate, especially in face-to-face interviews are keen to talk. Other methods could be used to complement the result.

It is essential to pay good attention not to transform the research into a study with a drug supply control focus: the research should have the scope to promote public health. Complementary methods and triangulation of data sources can be valuable to better understand the prison drug market in the current context, where new drugs are emerging and new methods of drug trafficking inside prison are identified. Participatory methods, where PLIP can provide their inputs, might support researchers in developing the study designs, as well as, in the dissemination of study results.

The paper highlights the importance of respecting high ethical standards while conducting prison research. This might represent an incentive for prison administrations which have never conducted drug research in prison, as it demonstrates, that facilitating access to prisons can produce useful information and practical benefits for prison professionals, for PLIP and the surrounding community. This consequently might decrease the fear of interference or negative impact of research on the prison administration, on PLIP or on the community.

The paper establishes a solid basis for the implementation of drug research in prison in Europe. The current evidence is mostly based on reviews and systematic reviews (Favril, 2024; Fazel and Seewald, 2012; Fazel *et al.*, 2016), which provide fundamental insights on the drug prevalence and related problems in the prison population.

This paper provides tools and practical advice based on practical multinational research experience (checklist, questionnaires, informed consent, etc.) that should facilitate direct research into prison. This can on the one hand promote the actual implementation of drug research in prison, and on the other hand it shows that conducting drug research is possible in several countries with different profiles, cultures and prison and/or drug regulations, producing evidence and ultimately benefits for all people living and working in prison.

## Conclusions

This paper provides a unique insight into recommended strategies to overcome challenges related to the implementation of ethical and robust drug research in prisons. It lays a foundation to support harmonised research about drugs in prison across countries.

## Supplementary Material

Data supplement 1

## Notes

[1.]
Link to the website of European Questionnaire on Drug Use among People living in prison (EQDP) (Long version)

[2.]
Link to the website of European Questionnaire on Drug Use among People living in prison (EQDP): Methodological guidelines

[3.] European Union Drugs Agency.

[4.]
Link to the website of European Questionnaire on Drug Use among People living in prison (EQDP): Methodological guidelines

## Supplementary material

The supplementary material for this article can be found online.
