# Supplementary material for: Conducting ethical drug research in prisons: lessons learnt from the implementation of the European Questionnaire on Drug use among People living in Prison (EQDP)
Source: Int J Prison Health (2024). 2026 Feb 2;22(2):303–15. doi: 10.1108/IJOPH-05-2025-0039 (PMC13701979; doi:10.1108/IJOPH-05-2025-0039)
Supplement: Supplementary file 1 [file ijoph-05-2025-0039_supplementary_file_1.docx]

**Supplemental File**

**Table 1. Check list for conducting drug research in European prisons**

**Table of content**

Preparation and planning	page 1

Methodological decisions	page 2

Implementation of EQDP studies	page 3

Adherence to ethical standards	page 4

**Supplemental File Table 1. Check list for conducting drug research in European prisons**

| **Area** | | **Actions** | | | **Tasks to check** | |  | |  |
| --- | --- | --- | --- | --- | --- | --- | --- | --- | --- |
| **Preparation and planning** | | | | | | | | |  |
| Planning | | Conceptualise and prepare the research in advance | | | Define research’s objectives and content | |  | |  |
|  |  | Timing | | | Start planning the research at least one year in advance of the actual foreseen implementation. | |  | |  |
|  |  | Mapping the prison system | | | Legislative framework, number and types of prison establishments, prison regimes and functioning. | |  | |  |
|  |  | Prepare a research protocol including objectives, method, variables, etc. | | | Prepare a Research Protocol including the tool to be used | |  | |  |
| Prison access | | Know how you can access prison in your country/region | | | Gather information on how to access prison for conducting drug research in your country | |  | |  |
|  |  | Achieve a deep knowledge of the prison system and context would help a better and speeder implementation of the research. | | | Map the actors inside prison | |  | |  |
|  |  | Obtain agreement with the relevant prison authorities for conducting drug research, following the rules established by the country and respecting the research protocol. | | | Send a formal request letter signed by the institutions responsible for the research, including research protocol (research objectives, methodology and process). Prepare a research protocol | |  | |  |
| Research staff | | Prepare a road map with roles, responsibilities and timing Identify human resources  Establish your study team, by identifying number and profile of researchers involved in data collection | | | Develop a roadmap of your work by identifying tasks and responsibilities for the different research phases | |  | |  |
|  |  |  |  |  | Identify the number of people you need for getting involved in the research in different phases, especially for the administration of the questionnaires. This could depend on the method used (self-administered, CAPI, face-to-face)- they can be internal or external to the institution responsible for the research | |  | |  |
|  |  |  |  |  | Choose researchers who fulfil the following requirements:   - do not work inside prison as prison staff. - have a scientific (not moralistic) attitude towards criminality and drug use. - demonstrate to be psychologically equipped to be confronted with difficult personal situations.   It might not be advisable to involve very unexperienced researchers, but this may depend on the context and the person | |  | |  |
|  |  |  |  |  |  | |  | |  |
|  |  |  |  |  | Conduct a short interview to select the research staff to be involved in data collection | |  | |  |
|  |  |  |  |  | Research staff should have an attitude that us respectful, not stigmatising, not judgemental, regardless the type of crime the person is in prison for | |  | |  |
|  |  |  |  |  | Provide scientific supervision to researchers administering the questionnaire | |  | |  |
|  |  |  |  |  | Provide training to staff involved in the research for the role they need to perform | |  | |  |
|  |  |  |  |  | Train the research on ethical considerations for conducting research in prison | |  | |  |
|  |  |  |  |  | Brief all research staff on prison security protocols | |  | |  |
|  |  | No conflict of interest should possible be included – if they exist, they must be disclosed | | | Disclose any conflict of interest as well as potential bias (constitution of the research team, academic/professional backgrounds of the researchers), potential cooperation with prison authorities | |  | |  |
|  |  | Emotional implications and attitudes | | | Consider the emotional implications that conducting research in prison may have on researchers and consequent impact on data collection | |  | |  |
|  | | Co-production is an asset when conducting drug research in prison: involving people with lived experience would help in better understanding the context and modalities for data collection and will enhance trustworthiness and credibility of the research in the community of people living in prison | | | If available, involve users’ association to identify a referent person for the implementation of the research  She/he can support the implementation of the survey  If not available, identify somebody after the first day of research, trying to consider her/his possible interest in the topic and the level of education/language spoken | |  | |  |
| Logistics | | Organise the logistics for conducting research in prison, considering the peculiar prison conditions (e.g. Internet, no pens, no copies to make, etc) | | | Identify and choose the space where you will be conducting the research in agreement with the prison administration- this may depend on the method of administration used | |  | |  |
|  |  |  |  |  | Choose a space that can host a number of persons adequate to your research method and the number of researchers involved | |  | |  |
|  |  |  |  |  | Choose a space that allows the people to respond to the survey anonymously | |  | |  |
|  |  |  |  |  | (if with pen and paper) Prepare sufficient paper copies of the questionnaires | |  | |  |
|  |  |  |  |  | (if with pen and paper and self-administered) Arrived equipped with a sufficient number of pens to be returned after the administration | |  | |  |
|  |  |  |  |  | If necessary, have a security staff present outside the space | |  | |  |
|  |  |  |  |  | Consider the time schedule of the daily life inside prison: meals, open air, work inside prison, etc.). | |  | |  |
|  |  |  |  |  | Be always prepared for unexpected changes | |  | |  |
| **Methodological decisions** | | | | | | | | |  |
| Research method | Choose the most appropriate method for your research purposes and objectives, following the EQDP methodological guidelines | | | If possible, choose a mixed method approach, which allows to combine quantitative and qualitative information, triangulating the different information sources. | | | |  | |
| Questionnaire | Use the EQDP to your research needs | | | Adapt the questionnaire to your research needs | | | |  | |
|  |  |  |  | Translate it if it is not available in your language (EQDP is already available in long and short versions in English, Croatian, French, German, Greek, Portuguese, Slovenian) | | | |  | |
|  |  |  |  | Always pay attention to the need for comparisons and harmonisation with other contexts: do not change wording- but you can shorten questions/group answers/ add questions | | | |  | |
|  |  |  |  | Pilot the questionnaire on a reasonable number of persons | | | |  | |
|  |  |  |  | In the methodological guidelines it is recommended to use a periodical cross-sectional survey (minimum every two years and maximum every four years) | | | |  | |
|  |  | | | Random sampling is recommended | | | |  | |
|  |  | | | Over-represent certain groups who may not be captured by a random sampling procedure (i.e. women, transgender people, foreign nationals, etc.) | | | |  | |
|  |  | | | Make all necessary efforts to always include female prisons or sections in your research | | | |  | |
| Sampling | Sampling is very important and should be clearly agreed with prison administration at the beginning of the survey, ensuring full independence of the researchers from the prison administration in the sampling choice | | | Define your sample: size and characteristics | | | |  | |
|  |  |  |  | Define the populations that are investigated, e.g. adolescents vs. adults; pre-trial vs. post-trial confinement; prison sentence vs. forensic commitment | | | |  | |
|  |  |  |  | Obtain a list of people living in prison at the time of the research: the list can be anonymous, but it is essential to define your sample and manage the research | | | |  | |
|  |  |  |  | A large proportion of PLIP have a language different from the language of the country where the prison is located: include information on languages spoken when you collect information on context, so you will know how many copies of the questionnaire in various languages you need to prepare | | | |  | |
| Administration mode | Choose the most appropriate mode of administration for your research purposes and objectives | | | Use the EQDP methodological guidelines to choose the most appropriate administration mode | | | |  | |
|  |  |  |  | Consider objectives, costs, logistic for choosing the best mode of administration | | | |  | |
|  |  |  |  | Self-administration should be preferred whenever possible | | | |  | |
|  |  |  |  | The level of illiteracy is high in prison: if a person cannot read and/or write be ready to administer the survey face to face | | | |  | |
|  |  |  |  | When you pilot the questionnaire, test also the most appropriate/effective method for your research purposes | | | |  | |
|  |  |  |  |  | | | |  | |
|  |  |  |  |  | | | |  | |
| Introduction | | Introduce the research to people who might participate and try to increase their participation | | | Introduce the research to people living in prison to be interviewed, explaining objectives, process and ethical measures | |  | |  |
|  |  | Incentives | | | Incentives for participating in the study/data collection can be used in agreement with the prison authorities. Those incentives should not include objects or goods that may put the people at any security and health risk (e.g., pens that can be used for drug injecting; money for internal transactions; etc.). | |  | |  |
| Nonparticipation | | In order to assess the representativeness of your sample it is important to achieve information on non-participation. | | | Record nonparticipation, if possible, including the reasons for non-participation (e.g. refusals, unavailability, sickness, work, etc | |  | |  |
| **Implementation of EQDP studies** | | | | | | | | | |
| Timing | | Consider the time when you can implement the research: | | | Agree with the prison administration the optimal time during the day to conduct the research. | |  | |  |
|  |  |  |  |  | Weekends may be preferable in some cases as people are not busy in several activities; however, in some context, the weekend may be more difficult as less custodial staff is available. This varies according to prison | |  | |  |
|  |  |  |  |  | Try to optimise the time, considering that time inside prison is slow | |  | |  |
|  |  |  |  |  |  | |  | |  |
|  |  |  |  |  |  | |  | |  |
| Presence of custodial staff | | Ensure independence from prison staff | | | Custodial staff should be at a distance that ensure full confidentiality and anonymity of the research | |  | |  |
| Feedback | | Discuss the research progress on a regular basis with the researchers, provide feedback to other stakeholders at the end of the research | | | At the end of each day identify a moment for an exchange of feedback on research administration among researchers  Organise sessions of feedback with people living in prison and prison administration to hear their opinion on the research, whether they found the results reflecting their context and conditions | |  | |  |
|  |  |  |  |  |  | |  | |  |
| Ad-hoc barriers to address | | Possible difficulties in collecting data in prison may arise from specific situation, like in the case of COVID19 | | | Consider specific contextual situations which may represent a specific difficulty and adopt a flexible approach to address it  Pay attention to not interfere with internal dynamics of formal and informal hierarchies both with people and staff | |  | |  |
|  |  |  |  |  |  |  |  | |  |
| Answering the questionnaire | | Facilitate respondents’ participation | | | Conduct the research in a context that can increase trustfulness, such as drug help centre if available or health prison services | |  | |  |
|  |  |  | | | In case you will use tablets, consider the available software to use questionnaires offline on a tablet and the possibility to synchronise when the questionnaire is completed and internet is available https://www.offlinesurveys.com/; https://www.project-redcap.org/ | |  | |  |
|  |  |  | | | In case of pen and paper, administer the questionnaire in small groups with assistance of a researcher | |  | |  |
|  |  | Adapt to the personal attitudes and skills of the respondents | | | Allow respondents time to respond at their own pace | |  | |  |
|  |  |  |  |  | Allow respondents to not answer certain questions. | |  | |  |
|  |  |  |  |  | Ensure that the existing relationship between the educator and the user does not bias the answers given by the latter. | |  | |  |
|  |  |  |  |  |  | |  | |  |
| Feedback | | Discuss the research progress on a regular basis with the researchers, provide feedback to other stakeholders at the end of the research | | | At the end of each day identify a moment for an exchange of feedback on research administration among researchers  Organise sessions of feedback with people living in prison and prison administration to hear their opinion on the research, whether they found the results reflecting their context and conditions | |  | |  |
| Presence of custodial staff | | Ensure independence from prison staff | | | Custodial staff should be at a distance that ensure full confidentiality and anonymity of the research | |  | |  |
| **Adherence to ethical standards** | | | | | | | | |  |
| Ethical standards | | | Set up ethical standards before the survey/data collection starts and maintain them during its implementation. | | | Rely on international guidelines including Declaration of Helsinki; the International Ethical Guidelines for Biomedical Research involving Human Subjects and the CIOMS guidelines for epidemiological studies  General Data Protection Regulations | |  | |
| Benefits for people | | | The survey should be carried out in a way that promotes its potential benefits for the people living in prison. People should not bear a disproportionate share of research burdens without a commensurate share of benefits | | | Ensure that the people are protected from any direct or indirect harms as consequences to participation | |  | |
|  |  |  |  |  |  | Inform people living in prison of their rights | |  | |
|  |  |  |  |  |  | Ensure that researchers ensure to inform people on the results of the survey | |  | |
|  |  |  |  |  |  | Include recommendations that aim to benefit people’s life inside prison in the final research results | |  | |
| Ethical approval | | | Obtain ethical approval | | | Obtain approval from ethical committee for conducting research in prison in your country | |  | |
|  |  |  |  |  |  | If such a committee does not exist, obtain ethical approval from the university or the institution under which the research is conducted | |  | |
| Confidentiality | | | Information should be anonymous to provide guarantees to PLIP | | | Ensure that the questionnaire is filled in in a fully anonymous way | |  | |
| Informed consent | | | Ensure the agreement of the participants | | | An informed consent should be obtained before starting the survey | |  | |
|  |  |  |  |  |  | A template is available | |  | |
| No obligation | | | The survey should be totally voluntary and people living in prison should provide informed true consent for their participation (see guidelines) | | | Explain people that they can refuse to participate at any stage of the research without any consequence | |  | |
